# The beneficial effects of a probiotic mix on bone and lean mass are dependent on the diet in female mice

**DOI:** 10.1038/s41598-025-91056-2

**Published:** 2025-02-20

**Authors:** Claes Ohlsson, Lina Lawenius, Yiwen Jiang, Karin Horkeby, Jianyao Wu, Karin H. Nilsson, Antti Koskela, Juha Tuukkanen, Sofia Movérare-Skrtic, Petra Henning, Klara Sjögren

**Affiliations:** 1 Department of Internal Medicine and Clinical Nutrition, Institute of Medicine, Sahlgrenska Osteoporosis Centre, Centre for Bone and Arthritis Research, Sahlgrenska Academy, University of Gothenburg, Gothenburg, Sweden; 2 Department of Anatomy and Cell Biology, Faculty of Medicine, Translational Medicine Research Unit, University of Oulu, Oulu, Finland

**Keywords:** Osteoporosis, Bone mass, Lean mass, Probiotic, Gut Microbiome, High-fat diet, Drug discovery, Microbiology, Physiology, Diseases, Endocrinology, Medical research

## Abstract

Bone mass and lean mass decrease with age and these changes are associated with increased fracture risk and sarcopenia. Previous studies demonstrated that a probiotic mixture of *Lacticaseibacillus paracasei* DSM13434, *Lactiplantibacillus plantarum* DSM 15312 and DSM 15313 (*L*. Mix) prevents bone loss in ovariectomized (ovx) female mice. The purpose of the present study is to test if the beneficial effect of *L*. Mix is modified by the diet. Female mice were fed either a high-fat (HFD, 60% kcal from fat) or a low-fat (LFD, 10% kcal from fat) diet and subjected to either sham or ovx surgery and treated with *L*. Mix for 12 weeks. *L*. Mix treatment increased total body bone mineral density (*p* ≤ 0.01), by increasing cortical bone area, and total body lean mass (*p* = 0.035) in mice on LFD but not in mice on HFD. Metagenome sequencing of cecal content showed that *L.* Mix treatment increased the relative abundance of *Lacticaseibacillus paracasei* and, *Lactiplantibacillus plantarum*, demonstrating successful treatment. In addition, the probiotic treatment affected the overall gut microbiota composition and functionality. These findings demonstrate that the *L.* Mix in combination with a healthy diet is beneficial for musculoskeletal health in female mice.

**Supplementary Information:**

The online version contains supplementary material available at 10.1038/s41598-025-91056-2.

## Introduction

Bone mass and lean mass decrease with age and these changes are associated with increased fracture risk and sarcopenia^1^, conditions that are associated with great suffering for patients and high costs for society^2^. There is a medical need to develop safe preventive treatments, to avoid fractures and sarcopenia. The gut microbiota (GM) can regulate bone mass in rodents and humans^3–8^ and it was recently demonstrated that certain gut microbiota species are associated with lean mass in a large human cohort^9^.

We have previously demonstrated that a probiotic mixture of *Lacticaseibacillus paracasei* DSM13434, *Lactiplantibacillus plantarum* DSM 15312 and DSM 15313 (*L*. Mix) prevents bone loss in ovariectomized (ovx) mice and postmenopausal women^4,7^. Diet is an important regulator of not only gut microbiota composition and functionality^10^, but also bone metabolism. High-fat diet causes obesity and studies in both humans^11–17^ and rodents^18–23^ have demonstrated that severe obesity reduces bone density and quality. However, it is unknown if the effects of diet on bone metabolism are modulated by the gut microbiota and if the effect of probiotics on bone mass is modulated by the dietary fat content^24^.

We recently showed that combining probiotic bacteria with dietary fiber protected against ovx-induced trabecular bone loss in a dose-dependent manner in mice^25^. Probiotic bacteria administered without a prebiotic fiber is dependent on the diet consumed by the host for fermentation and production of metabolites such as short chain fatty acids (SCFA) and amino acids^26,27^. Studies have demonstrated a bone protective effect of SCFA in germ free mice^28^ and ovx mice^29,30^. Amino acids have also been associated with bone health and Jennings, A. et al. demonstrated that amino acid intakes were positively associated with bone mineral density (BMD) in women^31^. In addition, we recently demonstrated that high circulating levels of the amino acid valine were associated with reduced hip fracture risk in humans^32^. Further studies are required to determine if the effects of dietary components on BMD are modulated by gut microbiota and if the effects of probiotics on BMD are modulated by dietary fiber or fat content.

Therefore, the aim of the present study was to test if the effects of *L*. Mix on bone mass and lean mass are modified by the dietary fat content. To this end, female sham or ovx mice were given a high-fat diet (HFD) with 60% of calories from fat or a control low-fat diet with 10% of calories from fat, matched for sucrose, vitamins and fiber content and treated with *L*. Mix or vehicle for 12 weeks.

## Results

### Treatment with a probiotic mix increased lean mass in female mice on LFD but not HFD

**Fig. 1:**
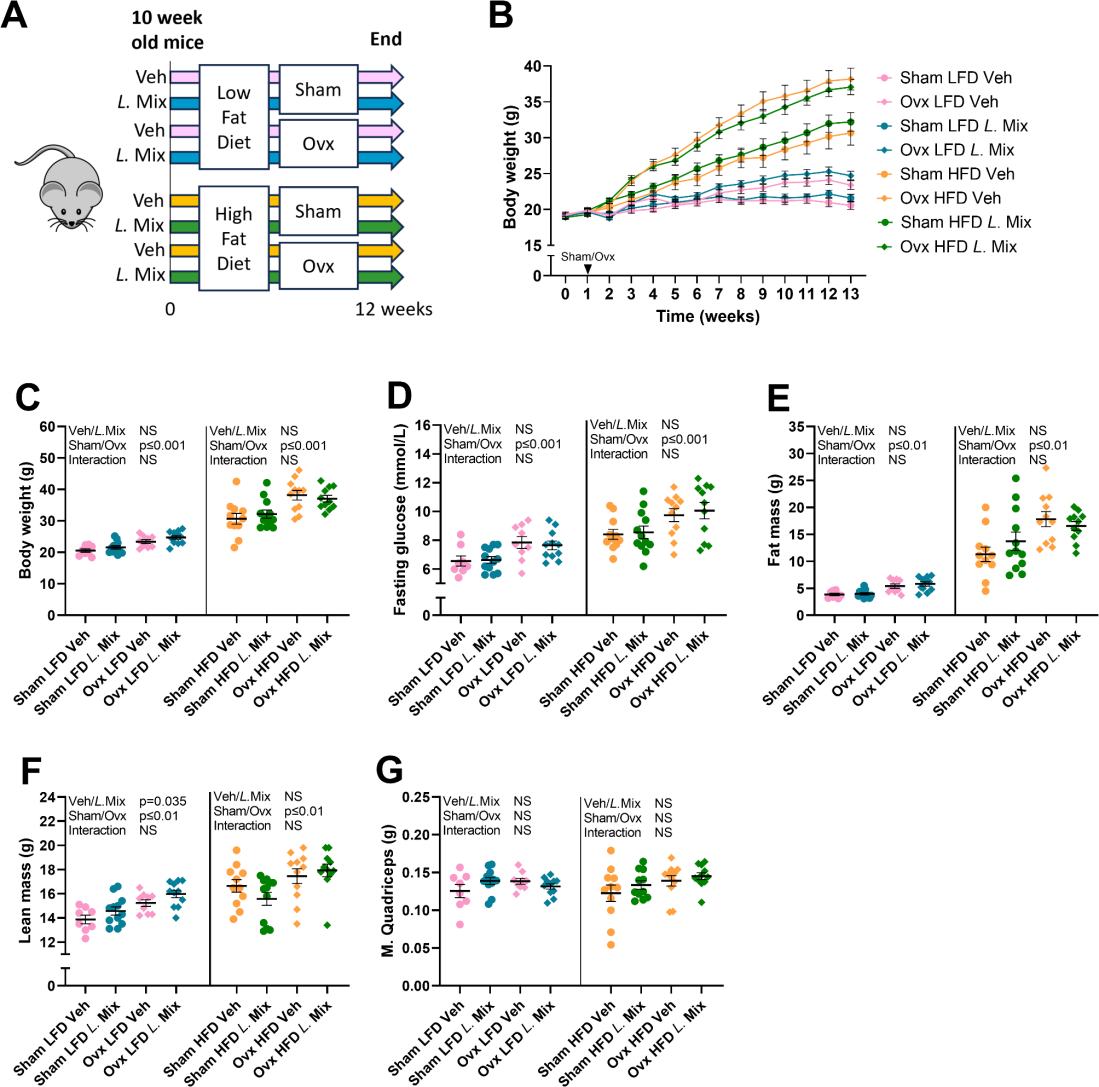
Treatment with a probiotic mix increased lean mass in mice on LFD but not HFD. Ten-week-old mice were subjected to either sham or ovariectomy (ovx) surgery and treated with a mixture of three probiotic bacteria (*L*. Mix) at a concentration of 10^9^ colony forming units/mL or vehicle in the drinking water for 12 weeks. Mice were fed a high-fat diet (HFD) with 60% kcal from fat (D12492, Research Diets) or a control low-fat diet (LFD) with 10% kcal from fat (D12450J). Overview of the experimental study design and groups of mice (A). Body weight during the study (B). Body weight (C), fasting glucose levels in serum (D), fat mass (E), lean mass (F), and weight of m. quadriceps (G) at the end of the study. Symbols in the scatter plots represent individual mice and the lines indicate mean ± SEM (*n* = 8–12). The overall effects of treatment (veh/*L.*Mix), surgical procedure (sham/ovx) and their interaction were calculated using two-way ANOVA in mice fed LFD and HFD separately (C, D, E, F, G). NS = not significant.

To test if the beneficial effects of the probiotic *L*. Mix on bone and body composition are affected by diet, ten-week-old mice were subjected to either sham or ovx surgery and treated with *L*. Mix or vehicle in the drinking water for 12 weeks. Mice were fed a HFD with 60% kcal from fat or a control LFD with 10% kcal from fat (Fig. 1A). The diets were matched for sucrose, vitamin and fiber content (Supplemental (S) Table 1). As expected, both HFD and ovx increased body weight, fat mass and fasting glucose levels (Fig. 1B-E). In contrast, treatment with the probiotic *L.* Mix had no effect on body weight, fat mass or fasting glucose levels (Fig. 1B-E). Interestingly treatment with the probiotic *L.* Mix increased total body lean mass (*p* = 0.035) in mice fed LFD but not HFD (Fig. 1F) although no significant effect was observed for the weight of m. quadriceps (Fig. 1G).

**Table 1:** Gut microbial species difference between mice treated with vehicle or *L*. Mix.

	*R*	*P*
**Sham Veh vs. Sham** ***L.*** **Mix**	0.490	0.001
**Ovx Veh vs. Ovx** ***L.*** **Mix**	0.337	0.003

Ten-week-old mice fed a low-fat diet (LFD) were subjected to either sham or ovariectomy (ovx) surgery and treated with a mixture of three probiotic bacteria (*L*. Mix) at a concentration of 10^9^ colony forming units/mL or vehicle in the drinking water for 12 weeks. The cecal microbial communities were analyzed by metagenome sequencing and the differences in gut microbiota species between the L. Mix treated and vehicle treated groups were analyzed by ANOSIM (*n* = 8–12). The R-value is a number between -1 and 1. A positive R value means that inter-group variation is larger than within group variation while 0 indicates that the between group variation and the within group variation is similar. The confidence degree is given by the P-value.

To summarize, treatment with the probiotic *L*. Mix increased lean mass in female mice fed LFD but not HFD.

### Treatment with a probiotic mix increased total body BMD via an effect on cortical bone area in mice fed LFD but not HFD

**Fig. 2:**
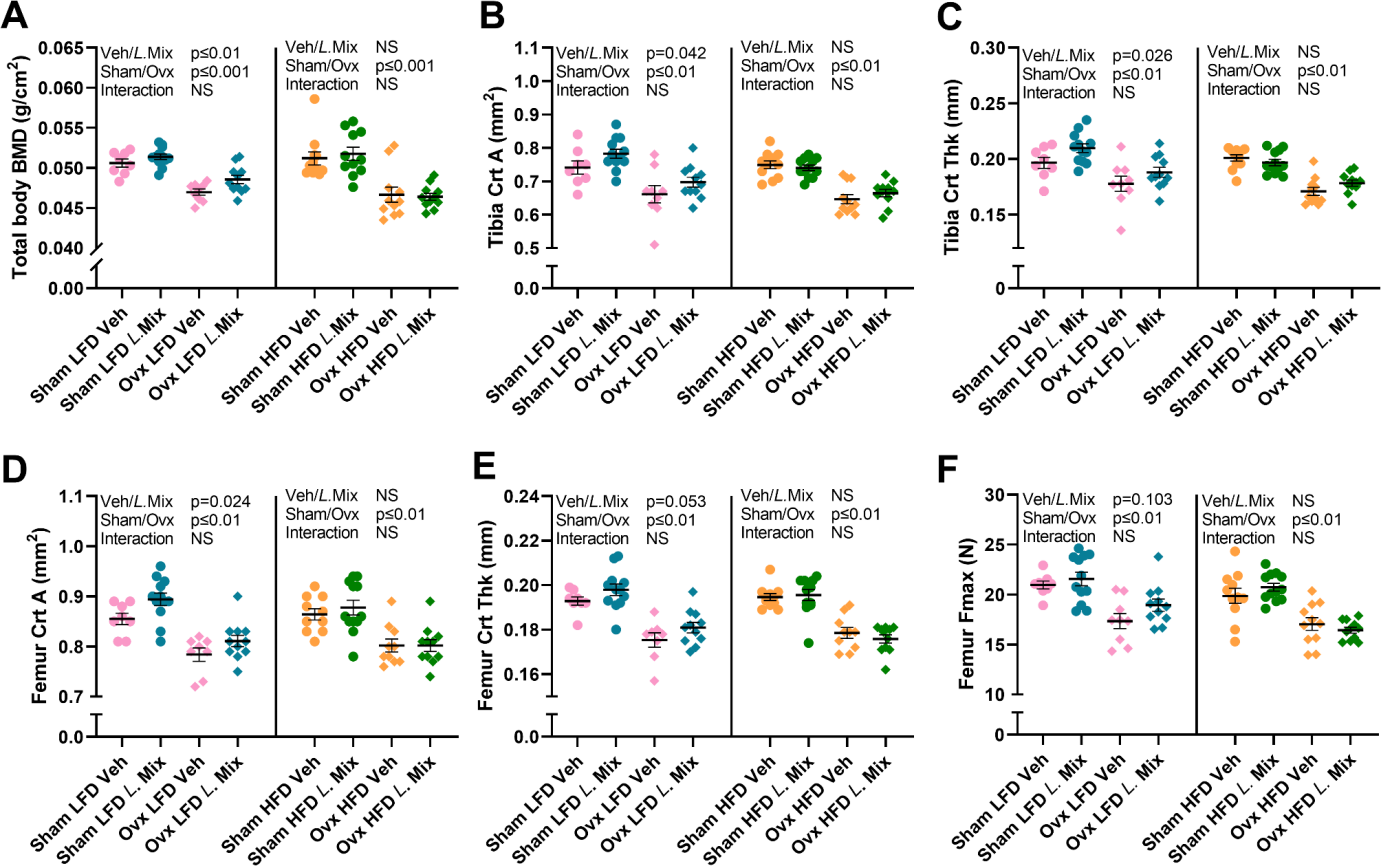
Treatment with a probiotic mix increased total body BMD via an effect on cortical bone mass in mice fed LFD but not HFD. Ten-week-old mice were subjected to either sham or ovariectomy (ovx) surgery and treated with a mixture of three probiotic bacteria (*L*. Mix) at a concentration of 10^9^ colony forming units/mL or vehicle in the drinking water for 12 weeks. Mice were fed a high-fat diet (HFD) with 60% kcal from fat (D12492, Research Diets) or a control low-fat diet (LFD) with 10% kcal from fat (D12450J). At the end of the study, mice were analyzed by DXA to determine the total body bone mineral density (BMD; A). Dissected tibias and femurs were analyzed with peripheral quantitative CT (pQCT) to measure cortical area (Crt A; B, D), and cortical thickness (Crt thk; C, E). The femur was analyzed by three-point bending to measure maximum force (Fmax; F). Symbols in the scatter plots represent individual mice and the lines indicate mean ± SEM (*n* = 8–12). The overall effects of treatment (veh/*L*.Mix), surgical procedure (sham/ovx) and their interaction were calculated using two-way ANOVA in mice fed LFD and HFD separately (C, D, E, F). NS = not significant.

Total body BMD measured by dual-energy x-ray absorptiometry (DXA) was decreased by ovx in mice on both LFD and HFD (Fig. 2A). Mice on LFD treated with the probiotic *L*. Mix had higher BMD compared to vehicle treated mice (Fig. 2A). In contrast, *L*. Mix had no effect on BMD in mice on HFD (Fig. 2A).

**Fig. 3:**
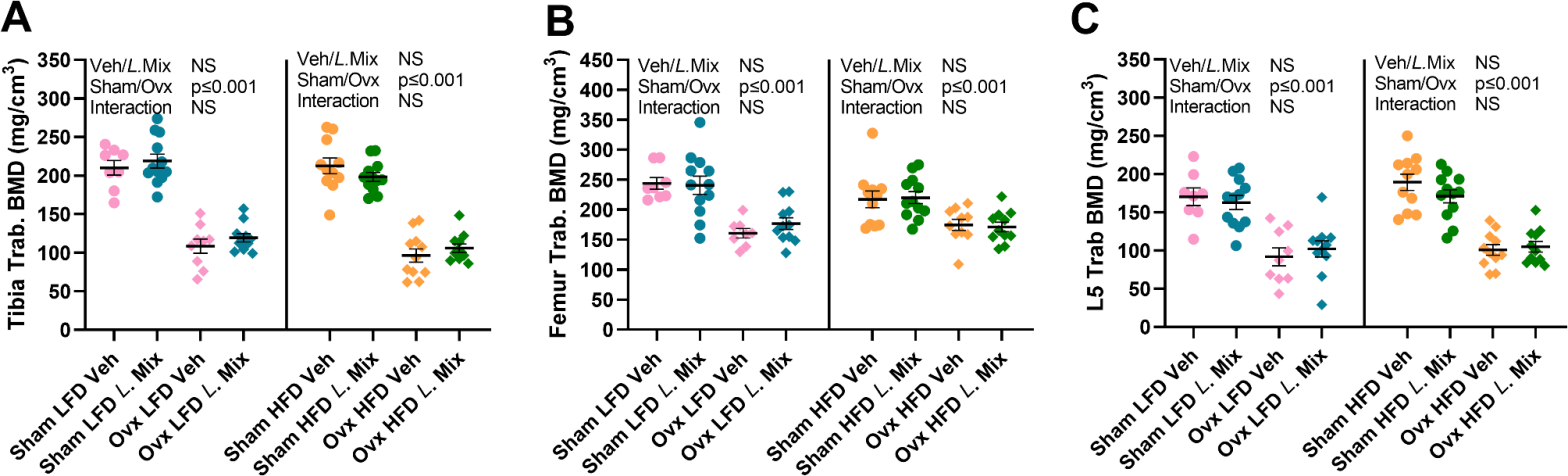
No effect of a probiotic mix on trabecular BMD. Ten-week-old mice were subjected to either sham or ovariectomy (ovx) surgery and treated with a mixture of three probiotic bacteria (*L*. Mix) at a concentration of 10^9^ colony forming units/mL or vehicle in the drinking water for 12 weeks. Mice were fed a high-fat diet (HFD) with 60% kcal from fat (D12492, Research Diets) or a control low-fat diet (LFD) with 10% kcal from fat (D12450J). At the end of the experiment, dissected tibias and femurs were analyzed with peripheral quantitative CT (pQCT) to measure trabecular bone mineral density (Trab. BMD; A, B) at the metaphyseal region. Lumbar vertebra 5 (L5) was analyzed with high-resolution microCT (µCT) to measure Trab. BMD (C). Symbols in the scatter plots represent individual mice and the lines indicate mean ± SEM (*n* = 8–12). The overall effects of treatment (veh/*L*.Mix), surgical procedure (sham/ovx) and their interaction were calculated using two-way ANOVA in mice fed LFD and HFD separately. NS = not significant.

A more detailed bone analysis using CT was done on dissected tibia and femur. As expected, ovx reduced both cortical and trabecular bone parameters at all bone sites examined in mice on both diets, but HFD compared to LFD had no impact on the magnitude of bone loss after ovx (Fig. 2, 3 S1-3). Treatment with *L.* Mix increased cortical area in both femur (Fig. 2D, *p* = 0.024) and tibia (Fig. 2B, *p* = 0.042) and cortical thickness in tibia (Fig. 2C, *p* = 0.026) with a similar trend in femur (Fig. 2E, *p* = 0.053) in mice on LFD but not on HFD (Fig. 2B-E). Bone strength was measured by three-point bending of femur, demonstrating a tendency for an increased maximum force (Fmax) of femur after *L*. Mix treatment in mice on LFD (*p* = 0.103, Fig. 2F). Treatment with *L*. Mix did not affect trabecular BMD in tibia, femur or vertebra (Fig. 3A-C).

The bone formation marker procollagen type I N-terminal propeptide (PINP) and the bone resorption marker collagen type I C-terminal telopeptides (CTX-I) were measured in serum collected at the end of the study but did not show any *L.* Mix effect (Figure S4). Analyses of gene expression of inflammatory cytokines in bone at the end of the study did not identify any effects of *L.* Mix on *Tnf-α*, *Il-6*, or *Il-1β* mRNA levels in bone (Figure S5). However, the expression of the early osteoblast-lineage marker *Runx2* in bone was increased by *L.* Mix treatment (Figure S5D) but *Opg*,* Rankl*,* Col1α1* and *Cathepsin K* expressions were unchanged (Figure S5).

Taken together, *L*. Mix increased total body BMD via an effect on cortical bone mass specifically in mice fed LFD.

### Treatment with the probiotic mix regulated the gut microbiota composition and functionality in mice fed LFD

**Fig. 4:**
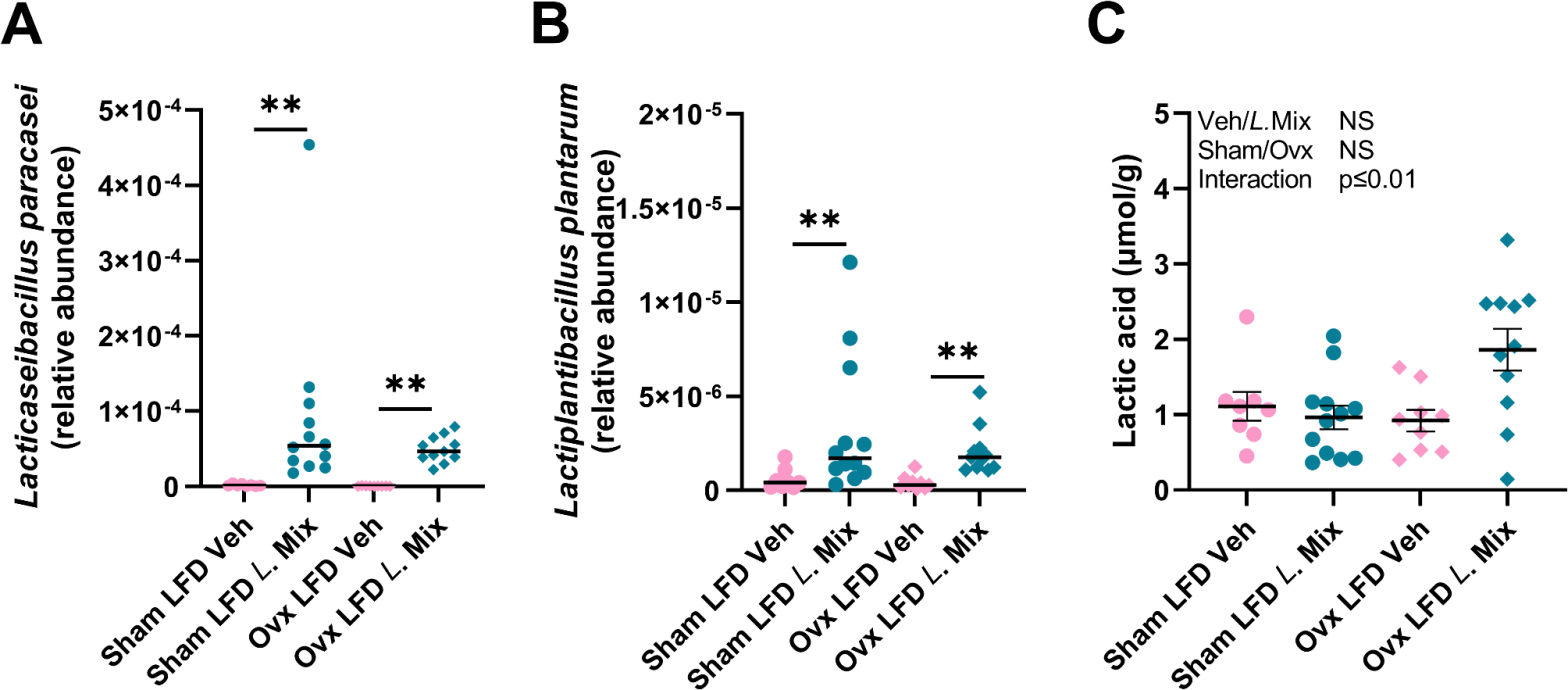
Lactic acid production and presence of probiotic bacteria in mice. Ten-week-old mice were subjected to either sham or ovariectomy (ovx) surgery and treated with a mixture of three probiotic bacteria (*L*. Mix) at a concentration of 10^9^ colony forming units/mL or vehicle in the drinking water for 12 weeks. Mice were fed a control low-fat diet (LFD) with 10% kcal from fat (D12450J, Research diets). Cecal samples were collected at the end of the study for metagenome sequencing to detect the presence of *Lacticaseibacillus paracasei* (A), *Lactiplantibacillus plantarum* (B) and to measure lactic acid by high performance liquid chromatography tandem mass spectrometry (UHPLC-MS/MS, C). Symbols in the scatter plots represent individual mice. For (A) and (B), the horizontal bars indicate median (*n* = 8–12) and the Kruskal-Wallis test was used, followed by Dunn´s post hoc test adjusted for multiple comparisons of *L.* Mix vs. veh in sham and ovx group respectively, ** *P* < 0.01. For (C), the overall effects of treatment (veh/*L*.Mix), surgical procedure (sham/ovx) and their interaction were calculated using two-way ANOVA (A). NS = not significant. The lines indicate mean ± SEM (*n* = 8–12).

We next determined the underlying mechanism for the stimulatory effect of *L*. Mix on BMD and lean mass in mice on LFD. To study the effect of *L*. Mix treatment on gut microbiota composition and functionality, we did metagenome sequencing of the cecal content. We started by analysing the cecal levels of the bacterial species present in *L*. Mix. The relative abundances of both *Lacticaseibacillus paracasei* and *Lactiplantibacillus plantarum* were low or undetectable in vehicle treated mice, while *L*. Mix treatment substantially increased the relative abundances of these two species (Fig. 4A, B), demonstrating that the *L*. Mix treatment was successful. Lactic acid is one of the major metabolites produced by *L.* Mix ^33^. There was a significant interaction term for the effects of *L*. Mix treatment and ovx on lactic acid in cecal content and subsequent post hoc analyses revealed that *L* Mix increased lactic acid in ovx mice (*p* < 0.01, Šídák’s multiple comparisons test) but not sham mice (Fig. 4C).

*L. Mix* treatment did not affect the alpha diversity as determined by Chao1, Simpson index or Shannon index (Figure S6 A-C).

**Fig. 5:**
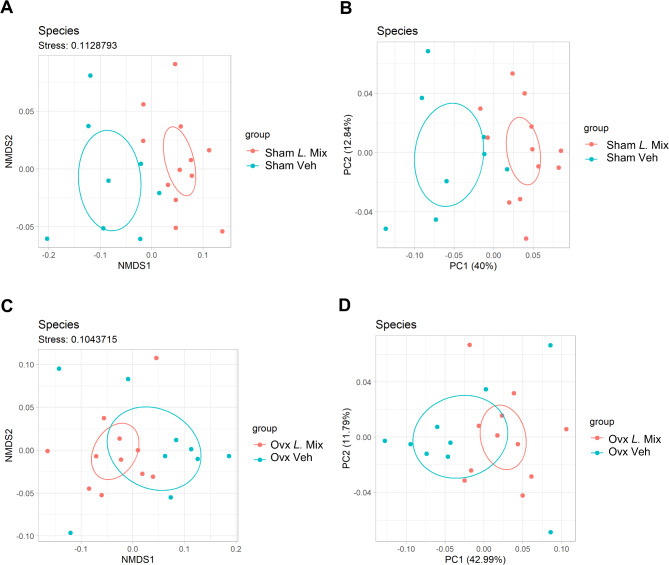
Treatment with the probiotic mix regulated the gut microbiota composition. Mice were subjected to either sham or ovariectomy (ovx) surgery, fed a low-fat diet (LFD) and treated with a mixture of three probiotic bacteria (*L*. Mix) at a concentration of 10^9^ colony forming units/mL or vehicle in the drinking water for 12 weeks. The cecal microbial communities at the species level were analyzed by metagenome sequencing (*n* = 8–12). Plots of the results from two-dimensional Non-metric Multidimensional Scaling (NMDS; A) and Principal Coordinate Analysis (PCoA; B) based on Bray-Curtis distance comparing *L.* Mix with veh treated sham mice. Plots of the results from two-dimensional NMDS (C) and PCoA (D) based on Bray-Curtis distance comparing *L.* Mix with veh treated ovx mice. The axes for the NMDS analysis show the scores of the individuals along the computed dimensions. The lower the stress value given above the plot, the better the NMDS analysis represents the underlying data. The percentages in parentheses represent the proportion of variation explained by the PCoA axes. Ellipses indicate 95% confidence intervals within groups.

There were no major overall differences between groups of the relative abundances at the phyla level (Figure S7A). However, statistical analyses of the relative abundance of the two most abundant phyla, *Bacillota* and *Bacteroidota*, showed that *L.* Mix decreased the relative abundance of *Bacillota* (Figure S7B) and increased the relative abundance of *Bacteroidota* (Figure S7C) in sham but not ovx mice, resulting in a decreased *Bacillota*/*Bacteroidota* ratio in *L*. Mix treated sham mice (Figure S7D).The analysis of similarities (ANOSIM) test was used to test if the overall gut microbiota composition at the species level was affected by the *L*. Mix treatment. There was a significant difference in species composition between the mice treated with *L*. Mix and veh in both sham and ovx mice (Table 1). The species differences between the *L.* Mix treated and vehicle treated groups were visualized by plotting the results from two-dimensional Non-metric Multidimensional Scaling (NMDS) analysis (Fig. 5A, C) and Principal Coordinate Analysis (PCoA) based on Bray-Curtis distance (Fig. 5B and D). Due to multiple testing challenges, the present study did not identify consistent treatment related changes in relative abundance of the individual species evaluated.

**Fig. 6:**
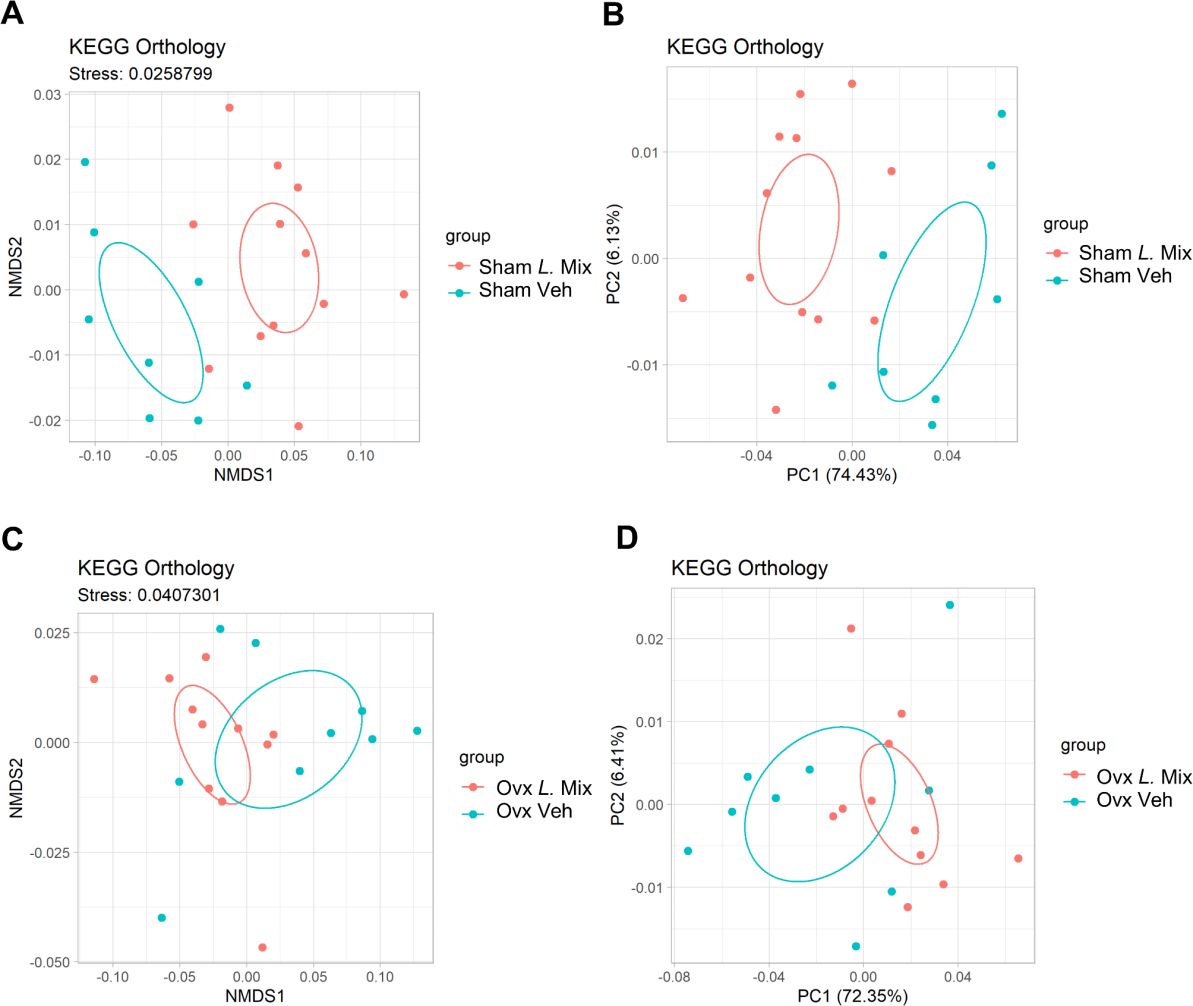
Treatment with the probiotic mix regulated the gut microbiota functionality. Mice were subjected to either sham or ovariectomy (ovx) surgery, fed a low-fat diet (LFD) and treated with a mixture of three probiotic bacteria (*L*. Mix) at a concentration of 10^9^ colony forming units/mL or vehicle in the drinking water for 12 weeks. The functionality of the gut microbiome was analyzed by metagenome sequencing at KEGG orthology level (*n* = 8–12). Plots of the results from two-dimensional Non-metric Multidimensional Scaling (NMDS; A) and Principal Coordinate Analysis (PCoA; B) based on Bray-Curtis distance comparing *L.* Mix with veh treated sham mice. Plots of the results from two-dimensional NMDS (C) and PCoA (D) based on Bray-Curtis distance comparing *L.* Mix with veh treated ovx mice. The axes for the NMDS analysis show the scores of the individuals along the computed dimensions. The lower the stress value given above the plot, the better the NMDS analysis represents the underlying data. The percentages in parentheses represent the proportion of variation explained by the PCoA axes. Ellipses indicate 95% confidence intervals within groups.

ANOSIM test of KEGG modules, Enzyme (ec) and KEGG orthology (ko) showed that the overall gut microbiota functionality differed between the *L.* Mix and veh treated groups in both sham and ovx mice (Table 2). The functional differences between the different groups at the KEGG orthology (ko) level were visualized by plotting the results from two-dimensional NMDS analysis (Fig. 6A and C) and PCoA based on Bray-Curtis distance (Fig. 6B and D).

**Table 2:** Functional difference in the gut Microbiome between mice treated with vehicle or *L*. Mix.

	*R*	*P*
**Module**		
Sham Veh vs. Sham *L.* Mix	0.439	0.001
Ovx Veh vs. Ovx *L.* Mix	0.127	0.048
**ec**		
Sham Veh vs. Sham *L.* Mix	0.5224	0.001
Ovx Veh vs. Ovx *L.* Mix	0.233	0.017
**ko**		
Sham Veh vs. Sham *L.* Mix	0.550	0.001
Ovx Veh vs. Ovx *L.* Mix	0.260	0.009

Ten-week-old mice fed a low-fat diet (LFD) were subjected to either sham or ovariectomy (ovx) surgery and treated with a mixture of three probiotic bacteria (*L*. Mix) at a concentration of 10^9^ colony forming units/mL or vehicle in the drinking water for 12 weeks. The cecal microbial communities were analyzed by metagenome sequencing and the differences in gut microbiota functionality (KEGG module, Enzyme (ec) and KEGG orthology (ko)) between the *L.* Mix treated and vehicle treated groups were analyzed by ANOSIM (*n* = 8–12). The R-value is a number between − 1 and 1. A positive R value means that inter-group variation is larger than within group variation while 0 indicates that the between group variation and the within group variation is similar. The confidence degree is given by the P-value.

To summarize, *L*. Mix treatment affected the gut microbiota composition and functionality.

## Discussion

The gut microbiota affects musculoskeletal health, and the gut microbiota is affected by the dietary fat content ^34^, but it is unknown whether the effects of the gut microbiota on musculoskeletal health are modified by the dietary fat content. We herein demonstrate that the beneficial effects of the probiotic *L*. Mix on BMD and lean mass are modified by the dietary fat content and that the beneficial effects by *L*. Mix treatment in mice on LFD are associated with changes in the gut microbiota composition and functionality. These findings demonstrate that the *L.* Mix treatment in combination with a healthy diet is beneficial for musculoskeletal health in mice.

We have earlier demonstrated that the *L.* Mix treatment increased total body lean mass in male mice on LFD (12% kcal from fat) but the possible modulatory role of the dietary fat content for this effect was not evaluated^35^. In the present study, we demonstrated that *L.* Mix increased total body lean mass in female mice on LFD but not on HFD. A role of the gut microbiota for lean mass regulation is supported by a large human observational study including more than 5000 participants, demonstrating that three gut microbiota species were positively associated with lean mass, also after adjustments for major known confounders such as the diet ^9^. However, the interaction between the diet and the gut microbiota composition for lean mass was not evaluated in that clinical study. Future well-powered clinical studies are warranted to determine the interaction between the dietary fat content and the gut microbiota composition for lean mass in humans.

We found no major effect of the HFD itself on BMD in the present study. Some previous studies suggesting a negative impact on bone parameters by HFD have used a diet not only high in fat content but also in sucrose^20,21^. Other studies provided the same type of HFD as used in the present study but did not give a control LFD that was matched for other nutrients ^22,36^. Chung E. et al. using HFD and LFD with similar compositions as that used in the present study observed a bone loss in HFD treated male mice^18^. However, neither of these studies investigated the interaction between the dietary fat content and the gut microbiota composition for the effect on bone mass. Importantly, while HFD alone did not impact BMD in the present study, our findings demonstrate that dietary fat content plays a role in modulating how probiotic treatment affects bone mass.

Detailed analyses using CT of excised long bones and vertebra revealed that the beneficial effect of *L.* Mix on BMD in mice on LFD was caused by an increased cortical cross sectional bone area while no significant effect was observed on trabecular bone mineral density. The relative importance of different beneficial probiotic treatments for the cortical vs. the trabecular bone compartments in mice has varied in previous studies and these differences may be due to different strains of mice used, sex differences, hormonal status, duration of treatment and/or interactions with dietary components^4, 35 ,37–40^.

In a recent clinical study postmenopausal women received two different doses of the probiotic *Limosilactobacillus reuteri* ATCC PTA 6475 (*L. reuteri*) or placebo daily for two years. Although no overall positive effect of this *L. reuteri* treatment was observed ^41^, a positive correlation between baseline BMI and the percent change in tibia BMD was observed both in the high-dose *L. reuteri* and in the low-dose *L. reuteri* groups but not in the placebo group, indicating an interaction between the *L. reuteri* treatment and baseline BMI for the change in BMD. However, the interactions between the diet and the probiotic-treatment on musculoskeletal health was not determined in that study.

Circulating bone turn-over markers analysed on serum collected at the end of the 12-week long study were not affected by *L*. Mix treatment, but it is possible that a new steady state of bone-turnover had been reached at this time point. The expression of the early osteoblast-lineage marker *Runx2* in bone was increased by *L.* Mix treatment, suggesting that the observed stimulatory effect of *L.* Mix on BMD may partly depend on recruitment of early osteoblast-lineage cells.

Metagenome sequencing of cecal content showed that *L.* Mix treatment substantially increased the relative abundances of *Lactiplantibacillus plantarum* and *Lacticaseibacillus paracasei* that are included in *L*. Mix, demonstrating successful delivery of the probiotic *L*. Mix treatment in the present study. Subsequent ANOSIM evaluation revealed that the overall gut microbiota composition at the species level, and functionality were affected by the *L*. Mix treatment in both sham and ovx mice. Due to multiple testing challenges and the use of only 8–12 mice in each experimental group, the present study did not have power enough to identify consistent treatment related changes in relative abundance of the individual species or of the individual KEGG module pathways evaluated. *Bacillota* and *Bacteroidota* are the phyla with the highest relative abundances in the present study and previous studies have reported that both ovariectomy and probiotic-treatment may regulate the *Bacillota/Bacteroidota* ratio ^42–44^. In the present study, we observed that the *Bacillota/Bacteroidota* ratio was reduced by *L.* Mix specifically in sham mice. As this decrease of the *Bacillota/Bacteroidota* ratio was only observed in the sham mice, we do not believe that this change is crucial for the bone-sparing effect of *L*. Mix, being similar in the sham and ovx mice. We propose that the observed beneficial effects of the *L*. Mix on musculoskeletal health may be either a direct effect exerted by the included *Lactiplantibacillus plantarum* and *Lacticaseibacillus paracasei* strains in *L.* Mix or an indirect effect because of an overall secondary change in the gut microbiota composition and functionality. Further mechanistic studies are required to explore the beneficial effect of *L.* Mix on musculoskeletal health and its interaction with the dietary fat content.

In conclusion, the beneficial effects of the probiotic *L.* Mix on bone and lean mass are dependent on the diet and are associated with changes in gut microbiota composition and functionality. These findings demonstrate that the *L.* Mix in combination with a healthy diet is beneficial for musculoskeletal health in female mice.

## Methods

### Mouse model, diet and treatment

C57BL/6J female mice purchased from Charles River (Germany) were housed in a standard animal facility under controlled temperature (22 °C), photoperiod (12 h light/dark cycle), with free access to fresh water, and pellet diet (Teklad diet 2016, Envigo). The mice were randomized into 8 groups (*n* = 12/group), with 4 mice/cage and acclimatized for two weeks to the animal facility. At 10 weeks of age, mice were subjected to ovx or sham surgery under anesthesia with isoflurane (Baxter Medical AB) and Meloxicam (Metacam, Boehringer Ingelheim Animal Health Nordics A/S) was given as postoperative analgesic. Mice were treated with either *Lacticaseibacillus paracasei* DSM13434 (L. paracasei 8700:2^®^), *Lactiplantibacillus plantarum* DSM 15312 (HEAL9^®^) and DSM 15313 (HEAL19) (*L.* Mix) proprietary strains of Probi AB, Sweden or vehicle (veh, maltodextrin) for 12 weeks starting at the day of ovx or sham surgery. The probiotic bacteria were given in the drinking water at a concentration of 10^9^ colony-forming units (cfu)/ml and water bottles were changed daily, similar as to our earlier studies^4,35,37^. Starting from surgery, the mice had free access to a high-fat diet (HFD) with 60% kcal from fat (D12492, Research Diets, New Brunswick, NJ) or a control low-fat diet (LFD) with 10% kcal from fat (D12450J, S Table 1). The following eight groups were included in the experiment: Four groups on LFD (Sham Veh, Ovx Veh, Sham *L*. Mix, Ovx *L*. Mix) and four groups on HFD (Sham Veh, Ovx Veh, Sham *L*. Mix, Ovx *L*. Mix, Fig. 1A). A few mice were removed during the study due to complications with the surgery or fighting, ending up with *n* = 8–12 in the groups. At the end of the study, following 3 h of food withdrawal, blood was taken from the tail tip to determine plasma glucose concentrations using an Accu-Check glucometer (Roche Diagnostics). Then, mice were anesthetized with Ketador/Dexdomitor (Richter Pharma/Orion Pharma), bled from the axillary vein, and thereafter euthanized by cervical dislocation. Blood was allowed to coagulate for at least 30 min at room temperature and centrifuged for 10 min, the serum was then collected and frozen. Tissues and cecal contents were snap frozen in liquid nitrogen. Bones were excised and fixed in 4% phosphate-buffered paraformaldehyde. All methods were performed in accordance with the relevant guidelines and regulations and experimental procedures involving animals were approved by the regional animal ethics committee in Gothenburg (ethics number: 4593/22). The study was performed and reported in accordance with ARRIVE guidelines.

## Dual X-ray absorptiometry

Total body BMD, fat mass, and lean mass were analyzed using Lunar PIXImus densitometer (Wipro GE Healthcare).

## Peripheral quantitative computed tomography (pQCT)

Peripheral quantitative computed tomography (pQCT; XCT Research M, Stratec Medizintechnik GmbH, Germany, resolution 70 µm^45^) was used to analyze the trabecular and cortical compartments of femur and tibia. Briefly, trabecular bone was analyzed in the metaphyseal region of the femur and tibia. The scan was positioned in the metaphysis at a distance corresponding to 3% of the total length from the distal growth plate in femur and 2.6% of the total length from the proximal growth plate in tibia and the trabecular bone region was defined by setting an inner area to 45% of the total cross-sectional area. The cortical bone was analyzed in the mid-diaphyseal region at 36% of the total length of the bone from the distal growth plate in femur and 30% of the total length from the proximal growth plate in tibia.

### High-resolution MicroCT (µCT)

High-resolution µCT analyses were performed using Skyscan 1275 scanner (Bruker MicroCT, Aartselaar, Belgium) as previously described^46^. Briefly, the L5 was imaged with an X-ray tube voltage of 50 kV, a current of 200 µA, and a 0.5 mm aluminium filter. The scanning angular rotation was 180° and the angular increment was 0.70°. The voxel size was 6.54 μm isotropically. NRecon (version 1.6.9) was used to perform the reconstruction after the scans. The trabecular bone in the vertebral body caudal of the pedicles was selected for analysis within a conforming volume of interest (cortical bone excluded) commencing at a distance 5 μm caudal of the lower end of the pedicles and extending a further longitudinal distance of 230 μm in the caudal direction.

## Biomechanics

The three-point bending test (span length 5.5 mm, loading speed 0.155 mm/s) was made at the mid femur using Instron universal testing machine (Instron 3366, Instron) after soaking the bones in PBS solution for 24 h. Based on the recorded load deformation curves, the biomechanical parameters were calculated from raw-data produced by Bluehill universal software v4.25 (Instron).

## Serum measurements

Enzyme immunoassay (EIA) kits were used to measure the bone resorption marker collagen type I C-terminal telopeptides (CTX-I; Immunodiagnostics Systems, Herlev, Denmark) and the bone formation marker procollagen type I N-terminal propeptide (P1NP; Immunodiagnostics Systems, Herlev, Denmark) in serum according to manufacturer’s directives.

### Gene expression in bone

Total RNA was prepared from cortical bone (femur with the ends removed and bone marrow flushed out with PBS before freezing) using TriZol Reagent (Sigma) followed by RNeasy Mini QIAcube kit (Qiagen). Real-Time PCR analyses were run using StepOnePlus Real-Time PCR systems (v2.3, Applied Biosystems). Predesigned probes for *Il1β* (Mm00434228_m1), *Rankl* (Mm00441908_m1), *Opg* (Mm00435452_m1), *Ctsk* (Mm00484036_m1), *Tnfα* (Mm00443258_m1), *Il-6* (Mm00446190_m1), *Col1α1* (Mm 00801666_g1), and *Runx2* (Mm00501580_m1) from Applied Biosystems were used. The mRNA abundance of each gene was calculated using the ΔΔCt method, adjusted for expression of *18 S ribosomal RNA* (4310893E, Applied Biosystems).

### Lactic acid

Lactic acid was measured by using a method based on derivatization of the analytes followed by quantitation by high performance liquid chromatography tandem mass spectrometry (UHPLC-MS/MS). Briefly, approximately 20 mg of each cecal sample was freeze dried and then resuspended in 1 mL water. The samples were derivatized as follows: 10 µL of each sample is added into a microcentrifuge tube followed by addition of 10 µL 75% methanol, 10 µL 200 mM 3-NPH (3-nitrophenylhydrazine in 75% MeOH) and 10 µL 120 mM EDC-6% pyridine (N-(3-Dimethylaminopropyl)-N-ethylcarbidiimide in 75% MeOH with 6% pyridine). The resulting mixture was mixed and incubated at room temperature for 45 min with shaking. The derivatization reaction was quenched by addition of 10 µL of 200 mM quinic acid in MeOH and the samples were mixed and incubated for 15 min at room temperature with shaking. 950 µL of water is added to the samples followed by mixing and centrifugation at 15 000 x g at room temperature for 5 min. 100 µL of each supernatant was then transferred to HPLC vials followed by addition of 100 µL of internal standard (13-C 3-nitrophenylhydrazine labeled short chain fatty acids). Samples were analyzed immediately after preparation by using a UHPLC-MS/MS consisting of an ExionLC UHPLC system coupled to a 6500 + QTRAP (both from AB Sciex LLC, Framingham, USA). The analytes were separated in a Phenomenex Kinetex C18 (100 × 2.1 mm, 1.7 μm, 100 Å) column, temperatured at 40 °C, by using the following gradient: 0–3 min 0.5% B, 3.00–3.01 min 0.5–2.5% B, 3.01–6.00 min 2.5–17% B, 6.00–10.00 min 17–45% B and 10.00–13.00 min 45–55% B, followed by washing and re-equilibration of the column. Mobile phase A and B were water and acetonitrile, respectively, and total flow was set to 0.4 mL/min. APCI ionization was used in positive polarity and the analytes were detected by using optimized MRM-transitions for each analyte and internal standard. A calibration curve covering the range of the analytes in the samples was injected together with the analytes.

### Statistical analyses

GraphPad Prism was used for all statistical analyses. Where indicated in the figure legend, results are presented as dot plots with lines representing means ± SEM. The overall effect of treatment (veh/*L*.Mix), surgical procedure (sham/ovx) and their interaction were calculated using two-way ANOVA. The interaction refers to difference in how the two groups, Veh/Sham respond to treatment with L. Mix and is given by the p-value for the interaction in the 2-way ANOVA analysis. When only two groups were compared, between-group differences were calculated using two-tailed Student’s *t-*test. *p* ≤ 0.05 was considered significant. For sub analyses of the metagenome data of phyla and bacterial species present in the probiotic *L.* Mix, results are presented as dot plots with bars representing medians as indicated in the figure legends. Kruskal-Wallis test was used, followed by Dunn´s post hoc test adjusted for multiple comparisons of *L.* Mix vs. Veh in sham and ovx group respectively.

### DNA extraction of cecal samples

Total DNA was prepared from cecal content using the QIAamp Fast DNA Stool kit (Qiagen, Hilden, Germany) according to the manufacturer’s recommendations for “Isolation of DNA from stool for pathogen detection”.

### Library construction, quality control and sequencing

All samples were paired-end sequenced on the Illumina NovaSeq x Plus platform (read length 150 bp) with a sequencing depth of 10 gigabases (Gb) raw data per sample at Novogene (UK) Company Limited. Before sequencing, **t**he genomic DNA was randomly sheared into short fragments and sequencing libraries were generated. The obtained fragments were end repaired, A-tailed and further ligated with Illumina adapter. The fragments with adapters were PCR amplified, size selected, and purified. The library was checked with Qubit and real-time PCR for quantification and bioanalyzer for size distribution detection. Quantified libraries were then pooled and sequenced.

### Bioinformatics analysis pipeline

#### Preprocessing of sequencing results

Fastp (https://github.com/OpenGene/fastp) was used for preprocessing raw data from the Illumina sequencing platform to obtain clean data for subsequent analysis. We discarded the paired reads in the following situation: when either one read contained adapter contamination, when either one read contained more than 10% uncertain nucleotides, when either one read contained more than 50% low quality nucleotides (base quality less than 5). To remove host contamination in samples, clean data was blasted to the host database to filter out reads that may come from host origin. Bowtie2 software (http://bowtie-bio.sourceforge.net/bowtie2/index.shtml) was used by default, with the following parameter settings: --end-to-end, --sensitive, -I 200, and -X 400 ^47–49^.

### Assembly of metagenome

MEGAHIT software was used for assembly analysis of clean data, with assembly parameter settings: --presets meta-large (--end-to-end, --sensitive, -I 200, -X 400) ^47,50^, and scaftigs without N was obtained by breaking the resulted scaffolds from the N junction ^51,52^.

### Gene prediction and abundance analysis

With the default parameters, MetaGeneMark (http://topaz.gatech.edu/GeneMark/) was used to perform ORF prediction for scaftigs ( > = 500 bp) of each sample^48,53–56^, and the information with a length less than 100 nt in the prediction results was filtered out. For the ORF prediction results, CD-HIT software (http://www.bioinformatics.org/cd-hit/) was used to eliminate redundancy ^57,58^ and obtain the non-redundant initial gene catalogue (the nucleic acid sequences encoded by successive non-redundant genes were called genes)^59^, with parameter settings: -c 0.95,-G 0,-aS 0.9,-g 1,-d 0^54,56^. Clean data of each sample was aligned to the initial gene catalogue by using Bowtie2 to calculate the number of reads of the genes on each sample alignment, with parameter settings: --end-to-end, --sensitive, -I 200, -x 400^51,54^. Genes with reads < = 2 in each sample were filtered out to finally determine the gene catalogue (Unigenes) for subsequent analysis^59^. Based on the number of reads aligned and the length of gene, the abundance of each gene in each sample was calculated ^60–62^. Based on the abundance of each gene in the gene catalogue in each sample, basic information statistics were performed.

### Species annotation

DIAMOND software (https://github.com/bbuchfink/diamond/)^61^ was used for alignment of unigenes sequences with Micro NR database, which includes sequences from bacteria, fungi, archaea, and viruses extracted from NCBI’s NR database (https://www.ncbi.nlm.nih.gov/). The alignment was performed using the blastp algorithm with a parameter setting of 1e-5^47^. From the alignment results of each sequence, the one with evalue < = min. evalue *10 was selected. Since each sequence may have multiple alignment results, LCA algorithm (applied to systematic taxonomy of MEGAN software (https://en.wikipedia.org/wiki/Lowest_common_ancestor) was adopted to determine the species annotation information of the sequence^63^.

Out of the results of LCA annotation and gene abundance table, the abundance of each sample at each taxonomy (kingdom, phylum, class, order, family, genus, or species) and the corresponding gene abundance tables were acquired. The abundance of a species in a sample was equal to the sum of the abundance of those genes annotated as that species ^48,54,64^. The number of genes of a species in a sample was equal to the number of genes whose abundance is non-zero among the genes annotated as that species. On the basis of the abundance tables at each taxonomy level, relative abundance overview were performed. Dimensionality reduction of the relative abundance measurements was performed using PCoA (function cmdscale in R), and NMDS (two dimensions, function metaMDS in R package vegan)^65^. Anosim analysis (R vegan package) was used to test the differences between groups. MetaGenomeSeq was used to search for species differences between groups. MetaGenomeSeq analysis was used to perform permutation test between groups on each taxonomy level and get a p-value.

### Annotations of common functional database

DIAMOND software (https://github.com/bbuchfink/diamond/) was used to align unigenes with those in the functional database, with parameter settings: blastp, -e 1e-5 ^54,64^. Functional databases include KEGG database (http://www.kegg.jp/kegg/)^66,67^. From the alignment results of each sequence, the best blast hit results were selected for subsequent analysis^54,56 ,68 ,69^. According to the alignment results, the relative abundance at different functional levels was calculated (the relative abundance at each functional level is equal to the sum of the relative abundance of genes annotated as that functional level) ^48,54^. The gene number table of each sample at each taxonomy level was derived from the result of functional annotation and gene abundance table. The number of genes with a certain function in a sample is equal to the number of genes whose abundance is non-zero among the genes annotated with this function. Based on the abundance table at each taxonomy level, annotated genes statistics, relative abundance overview, and abundance clustering heat map were carried out, combined with PCoA and NMDS, Anosim analysis of inter-/intra-group differences on the basis of functional abundance and MetaGenomeSeq on inter-group functional difference.

## Electronic supplementary material

Below is the link to the electronic supplementary material.

Supplementary Material 1

## Data Availability

The sequencing files are deposited at sequencing read archive (SRA) at NCBI, accession number: PRJNA1187318.
