# Supplementary material for: The beneficial effects of a probiotic mix on bone and lean mass are dependent on the diet in female mice
Source: Sci Rep. 2025 Feb 20;15:6182. doi: 10.1038/s41598-025-91056-2 (PMC11842756; doi:10.1038/s41598-025-91056-2)
Supplement: Supplementary file 1 — Supplementary Material 1 [file 41598_2025_91056_MOESM1_ESM.docx]

**Supplemental (S) material**

**Table S 1.**

| Class description | Ingredients | D12492  60 kcal% Fat | D12450J  10 kcal% Fat |
| --- | --- | --- | --- |
| Protein | Casein, Lactic, 30 Mesh | 200.00 g | 200.00 g |
| Protein | Cystine, L | 3.00 g | 3.00 g |
| Carbohydrate | Starch, Corn |  | 506.20 g |
| Carbohydrate | Lodex 10 | 125.00 g | 125.00 g |
| Carbohydrate | Sucrose, Fine Granulated | 72.80 g | 72.80 g |
| Fiber | Solka Floc, FCC200 | 50.00 g | 50.00 g |
| Fat | Lard | 245.00 g | 20.00 g |
| Fat | Soybean Oil, USP | 25.00 g | 25.00 g |
| Mineral | S10026B | 50.00 g | 50.00 g |
| Vitamin | Choline Bitartrate | 2.00 g | 2.00 g |
| Vitamin | V10001C | 1.00 g | 1.00 g |
| Dye | Dye, Blue FD&C #1, Alum. Lake 35-42% | 0.05 g | 0.01 g |
| Dye | Dye, Yellow FD&C #5, Alum. Lake 35-42% |  | 0.04 g |
|  | Total: | 773.85 g | 1055.05 g |

Diet formulas for high-fat diet HFD with 60% kcal from fat (D12492, Research Diets) and control low-fat diet with 10% kcal from fat (D12450J).


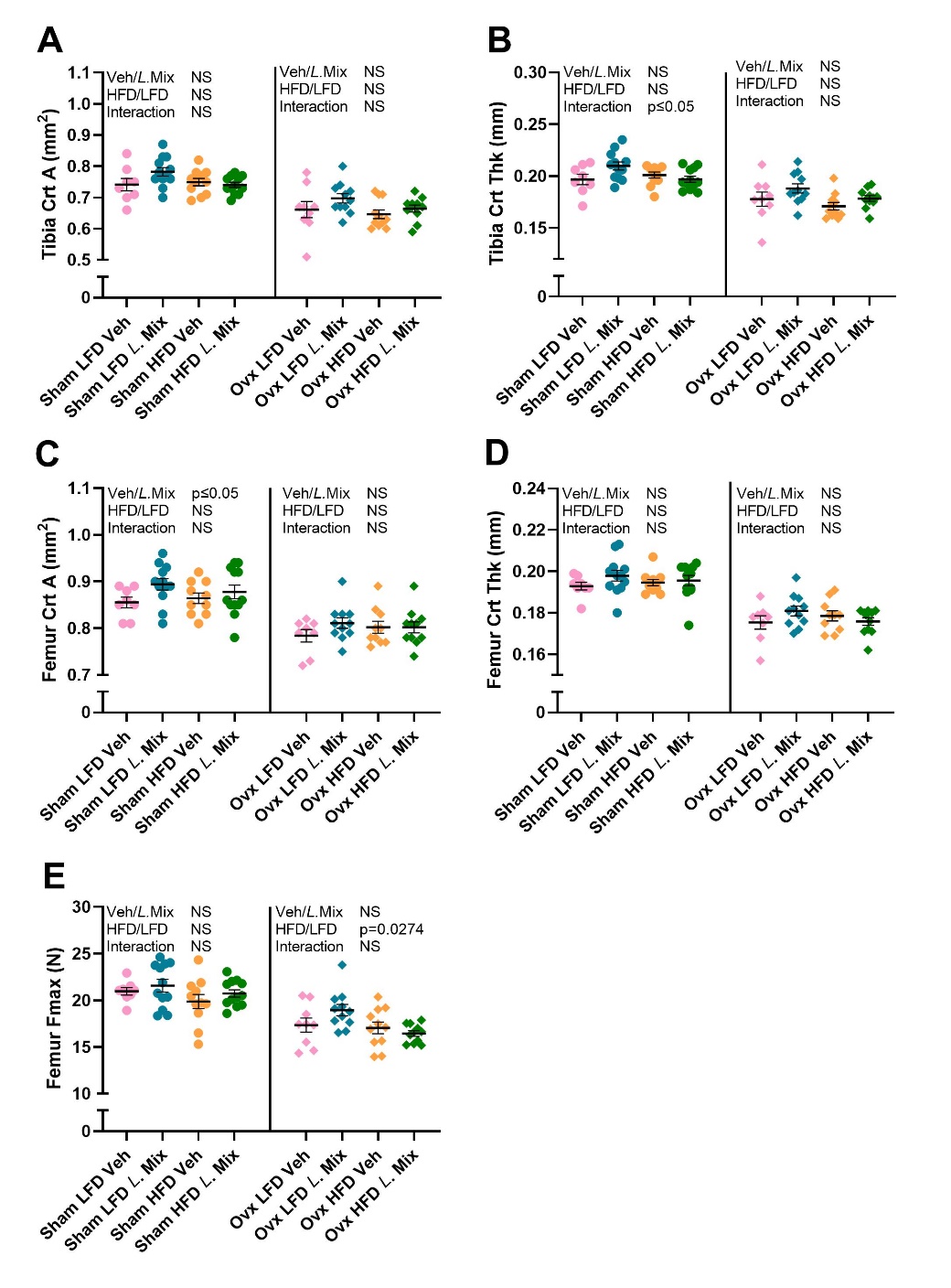


**Figure S1. No major effect of HFD on cortical bone in either sham or ovx mice**

Ten-week-old mice were subjected to either sham or ovariectomy (ovx) surgery and treated with a mixture of three probiotic bacteria (*L*. Mix) at a concentration of 10^9^ colony forming units/mL or vehicle in the drinking water for 12 weeks. Mice were fed a high-fat diet (HFD) with 60% kcal from fat (D12492, Research Diets) or a control low-fat diet (LFD) with 10% kcal from fat (D12450J). At the end of the study, dissected tibias and femurs were analyzed with peripheral quantitative CT (pQCT) to measure cortical area (Crt A; A, C), and cortical thickness (Crt thk; B, D). The femur was analyzed by three-point bending to measure maximum force (Fmax; E). Symbols in the scatter plots represent individual mice and the lines indicate mean±SEM (n=8-12). The overall effects of treatment (veh/*L*.Mix), diet (HFD/LFD) and their interaction were calculated using two-way ANOVA in sham and ovx mice separately. NS=not significant.

**
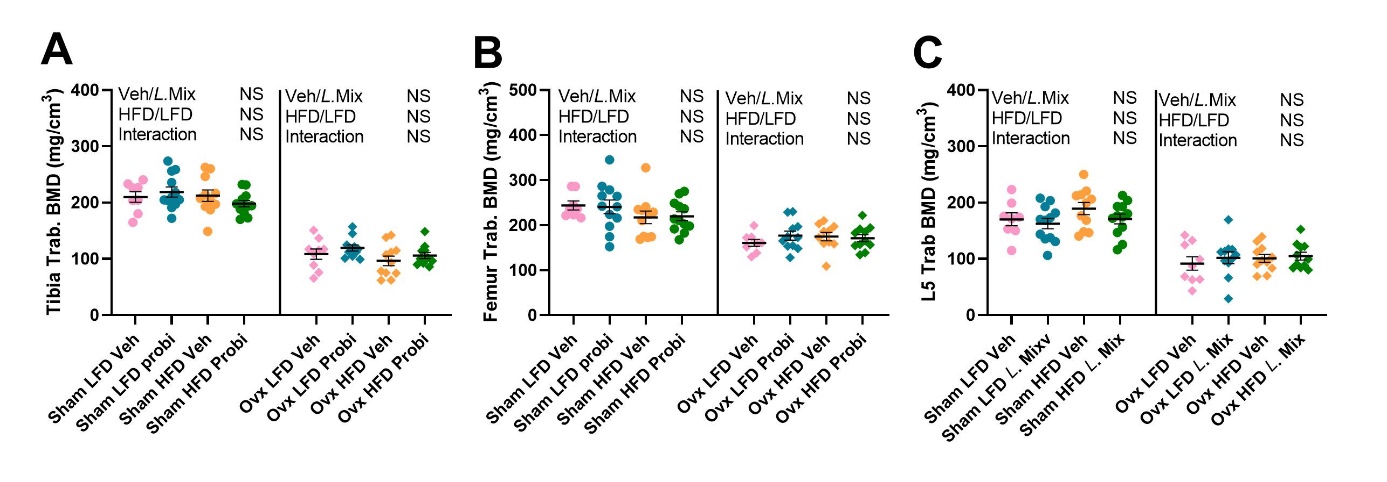
**

**Figure S2. No effect of HFD on trabecular bone in either sham or ovx mice.**

Ten-week-old mice were subjected to either sham or ovariectomy (ovx) surgery and treated with a mixture of three probiotic bacteria (*L*. Mix) at a concentration of 10^9^ colony forming units/mL or vehicle in the drinking water for 12 weeks. Mice were fed a high-fat diet (HFD) with 60% kcal from fat (D12492, Research Diets) or a control low-fat diet (LFD) with 10% kcal from fat (D12450J). At the end of the experiment, dissected tibias and femurs were analyzed with peripheral quantitative CT (pQCT) to measure trabecular bone mineral density (Trab. BMD; A, B) at the metaphyseal region. Lumbar vertebra 5 (L5) was analyzed with high-resolution microCT (µCT) to measure Trab. BMD (C). Symbols in the scatter plots represent individual mice and the lines indicate mean±SEM (n=8-12). The overall effects of treatment (veh/*L*.Mix), diet (HFD/LFD) and their interaction were calculated using two-way ANOVA in sham and ovx mice separately. NS=not significant.


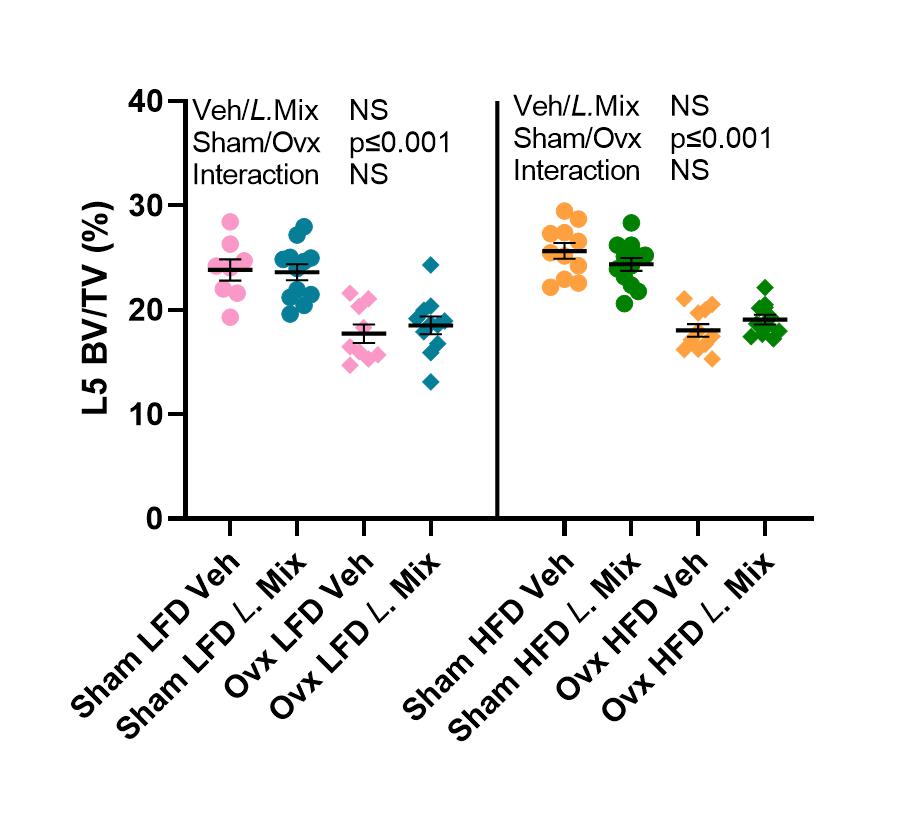


**Figure S3. Trabecular bone volume fraction of the lumbar vertebra**

Ten-week-old mice were subjected to either sham or ovariectomy (ovx) surgery and treated with a mixture of three probiotic bacteria (*L*. Mix) at a concentration of 10^9^ colony forming units/mL or vehicle in the drinking water for 12 weeks. Mice were fed a high-fat diet (HFD) with 60% kcal from fat (D12492, Research Diets) or a control low-fat diet (LFD) with 10% kcal from fat (D12450J). At the end of the experiment, lumbar vertebra 5 (L5) was analyzed with high-resolution microCT (µCT) to measure trabecular bone volume fraction (BV/TV). Symbols in the scatter plots represent individual mice and the lines indicate mean±SEM (n=8-12). The overall effects of treatment (veh/*L*.Mix), surgical procedure (sham/ovx) and their interaction were calculated using two-way ANOVA in mice fed LFD and HFD separately. NS=not significant.

**
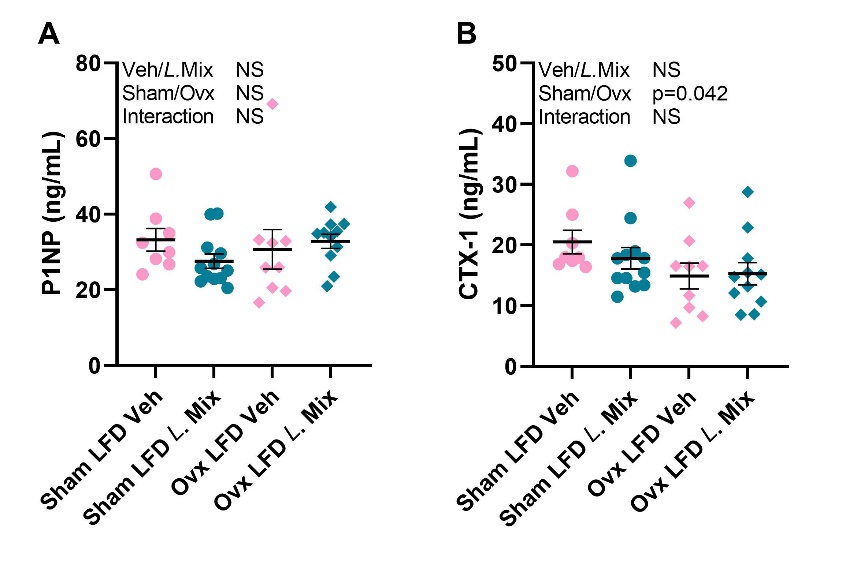
**

**Figure S4. Bone turnover markers.**

Ten-week-old mice fed a low-fat diet (LFD) were subjected to either sham or ovariectomy (ovx) surgery and treated with a mixture of three probiotic bacteria (*L*. Mix) at a concentration of 10^9^ colony forming units/mL or vehicle in the drinking water for 12 weeks. At the end of the experiment, the bone formation marker procollagen type I N-terminal propeptide (PINP; A) and the bone resorption marker collagen type I C-terminal telopeptides (CTX-I; B) were measured in serum. Symbols in the scatter plots represent individual mice and the lines indicate mean±SEM (n=8-12). The overall effects of treatment (veh/*L*.Mix), surgical procedure (sham/ovx) and their interaction were calculated using two-way ANOVA. NS=not significant.

**
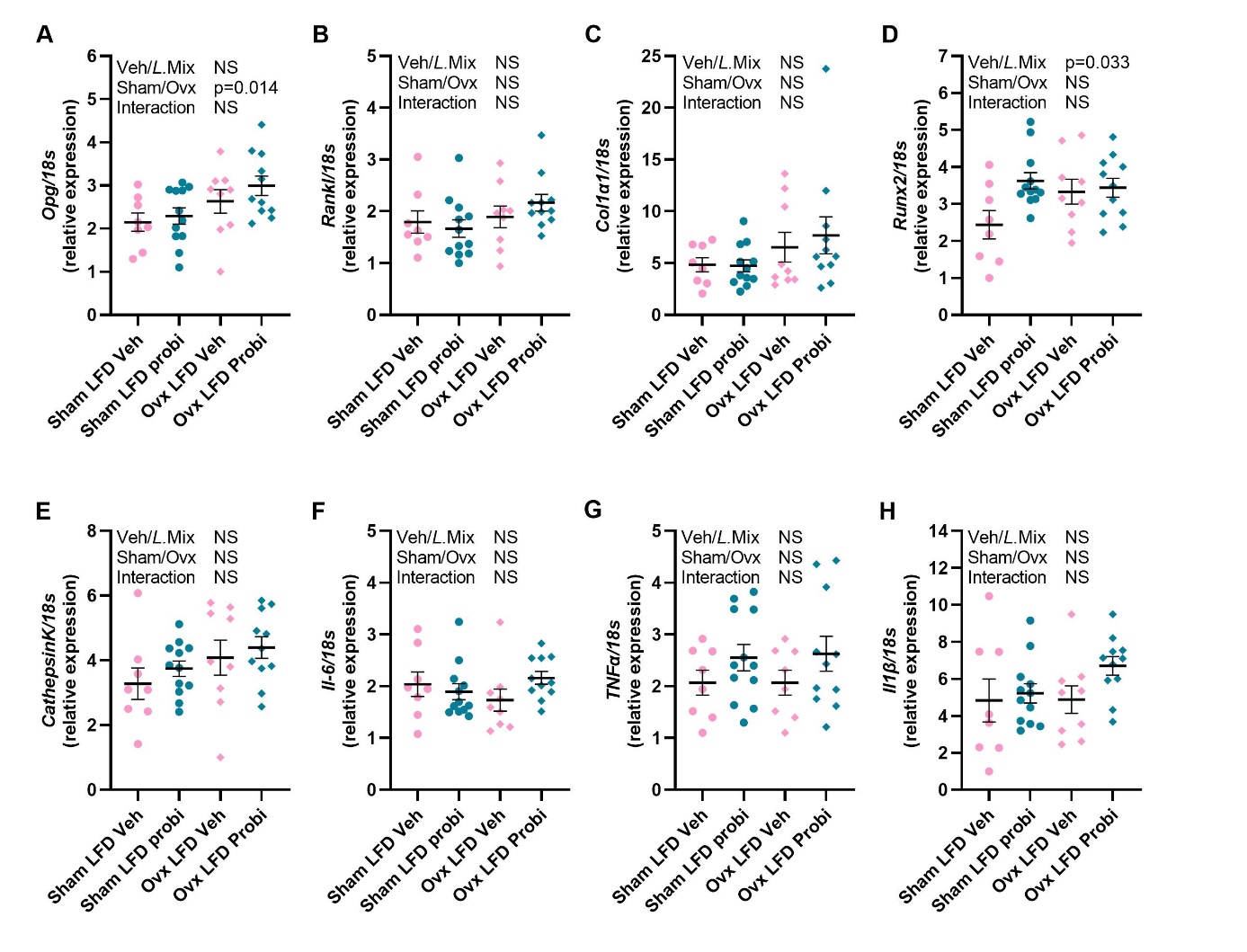
Figure S5. Gene expression in femur cortical bone.**

Ten-week-old mice fed a low-fat diet (LFD) were subjected to either sham or ovariectomy (ovx) surgery and treated with a mixture of three probiotic bacteria (*L*. Mix) at a concentration of 10^9^ colony forming units/mL or vehicle in the drinking water for 12 weeks. RNA was prepared from femur cortical bone collected at the end of the experiment to measure the gene expression of *Opg* (A)*, Rankl* (B)*, Col1α1* (C)*, Runx2* (D)*, Cathepsin K* (E)*, Il-6* (F)*, Tnf-α* (G) and *Il-1β* (H). Symbols in the scatter plots represent individual mice and the lines indicate mean±SEM (n=8-12). The overall effects of treatment (veh/*L*.Mix), surgical procedure (sham/ovx) and their interaction were calculated using two-way ANOVA. NS=not significant.


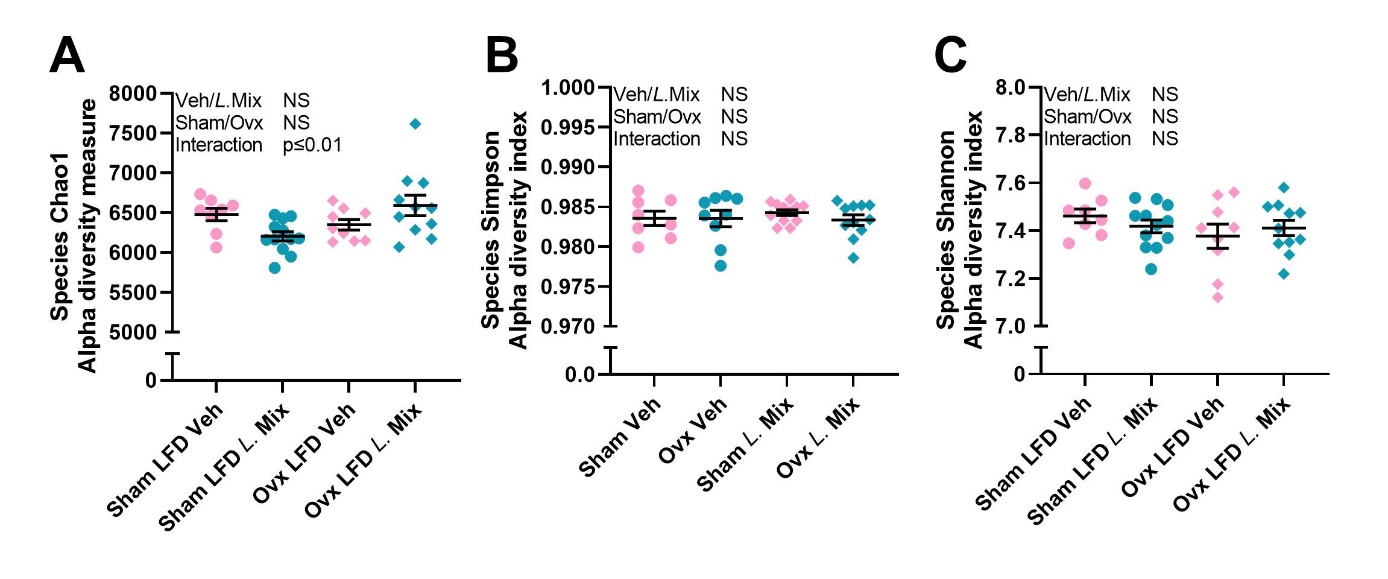


**Figure S6. Gut microbiome, alpha diversity**

Ten-week-old mice fed a low-fat diet (LFD) were subjected to either sham or ovariectomy (ovx) surgery and treated with a mixture of three probiotic bacteria (*L*. Mix) at a concentration of 10^9^ colony forming units/mL or vehicle in the drinking water for 12 weeks. The cecal microbial communities were analyzed by metagenome sequencing and alpha diversity was determined by Chao1 (A), Simpson (B), and Shannon (C). Symbols in the scatter plots represent individual mice and the bars indicate mean±SEM (n=8-12). The overall effects of treatment (veh/*L*.Mix), surgical procedure (sham/ovx) and their interaction were calculated using two-way ANOVA in mice fed LFD. NS=not significant.


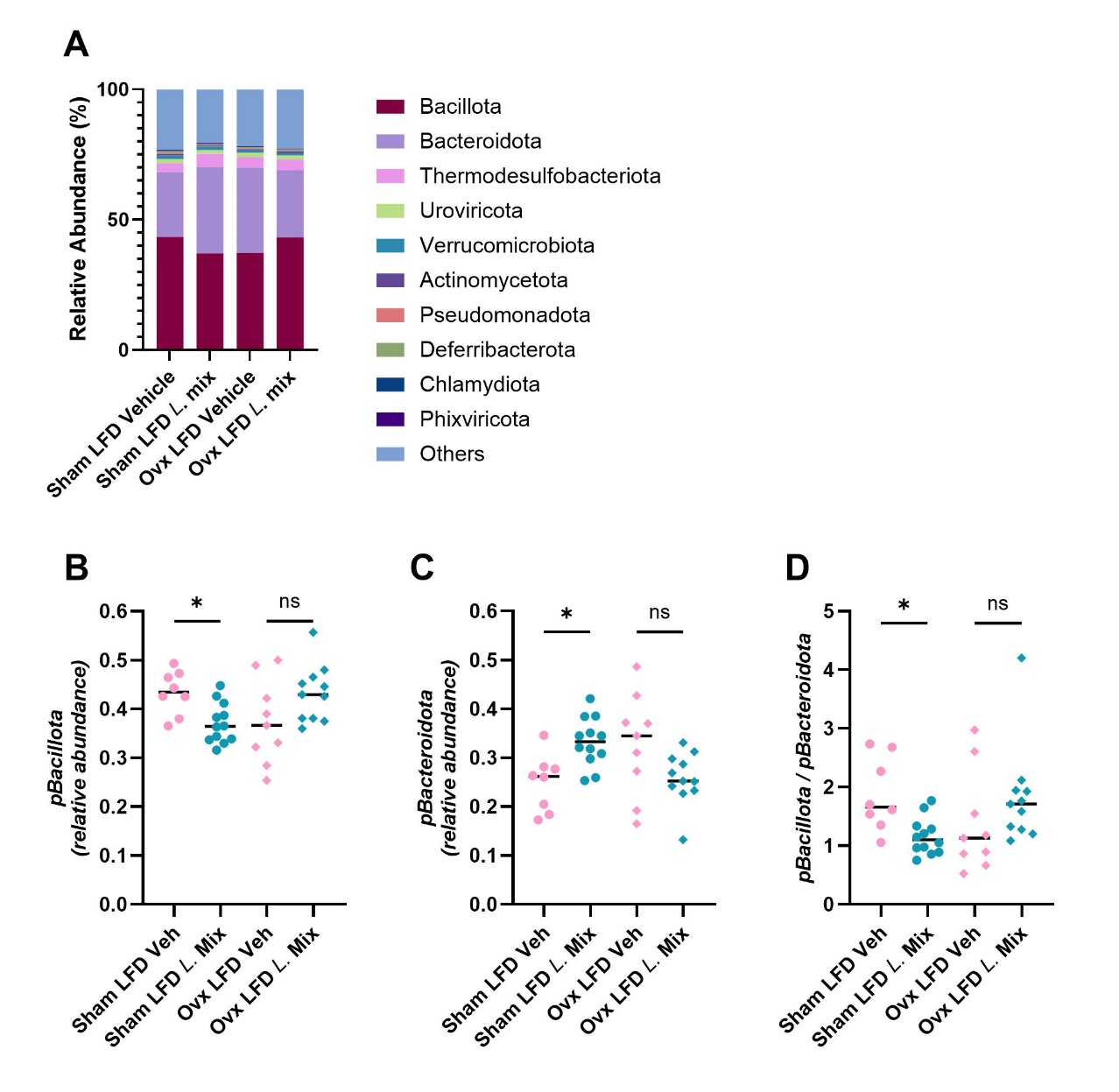


**Figure S7. Relative phyla levels and *Bacillota/Bacteroidota* ratio**

Ten-week-old mice fed a low-fat diet (LFD) were subjected to either sham or ovariectomy (ovx) surgery and treated with a mixture of three probiotic bacteria (*L*. Mix) at a concentration of 10^9^ colony forming units/mL or vehicle in the drinking water for 12 weeks. Cecal samples were collected at the end of the study for metagenome sequencing. The relative abundances at the phyla level (A), the relative abundance of *Bacillota* (B)*, Bacteroidota* (C) *and Bacillota/Bacteroidota* ratio (D) were analyzed. Symbols in the scatter plots (B-D) represent individual mice and the horizontal bars indicate median (n=8-12). The Kruskal-Wallis test were used, followed by Dunn´s post hoc test adjusted for multiple comparisons of *L.* Mix vs Veh in sham and ovx group respectively (B_D), * P<0.05.
